# Early signs of atherosclerosis are associated with insulin resistance in non-obese adolescent and young adults with type 1 diabetes

**DOI:** 10.1186/1475-2840-11-145

**Published:** 2012-11-27

**Authors:** Björn Rathsman, Stefan Rosfors, Åke Sjöholm, Thomas Nyström

**Affiliations:** 1 Karolinska Institutet, Department of Clinical Science and Education, Sachs’ Childrens’ Hospital, Södersjukhuset AB, Stockholm, SE-118 83, Sweden; 2 Karolinska Institutet, Department of Clinical Science and Education, Section of Clinical Physiology, Södersjukhuset AB, Stockholm, Sweden; 3 Karolinska Institutet, Department of Clinical Science and Education, Division of Internal Medicine, Södersjukhuset AB, Stockholm, Sweden

**Keywords:** Adolescent, Atherosclerosis, Carotid intima-media thickness, Insulin sensitivity, Type 1 diabetes

## Abstract

**Background:**

Patients with type 1 diabetes have a substantial risk of developing cardiovascular complications early in life. We aimed to explore the role of insulin sensitivity (S_i_) as an early factor of atherosclerosis in young type 1 diabetes *vs.* non-diabetic subjects.

**Methods:**

Forty adolescent and young adult individuals (20 type 1 diabetics and 20 non-diabetics), age 14–20 years, without characteristics of the metabolic syndrome, participated in this cross-sectional study. After an overnight fast, S_i_ was measured by hyperinsulinemic euglycemic clamp (40 mU/m^2^) and calculated by glucose infusion rate (GIR). Carotid intima-media thickness (cIMT) was measured in the common carotid artery with high-resolution ultrasonography. Risk factors of atherosclerosis (Body mass index [BMI], waist circumference, systolic blood pressure [sBP], triglycerides, low HDL-cholesterol and HbA_1c_) were also investigated.

**Results:**

cIMT was increased (0.52 ± 0.1 *vs.* 0.47 ± 0.1 mm, *P* < 0.01), whereas GIR was decreased (5.0 ± 2.1 *vs.* 7.1 ± 2.2 mg/kg/min, *P* < 0.01) in type 1 diabetics *vs.* non-diabetics. The differences in cIMT were negatively associated with S_i_ (*r* = −0.4, *P* < 0.01) and positively associated with waist circumference (*r* = 0.34, *P* = 0.03), with no such associations between BMI (*r* = 0.15, *P* = 0.32), sBP (*r* = 0.09, *P* = 0.58), triglycerides (*r* = 0.07, *P* = 0.66), HDL-cholesterol (*r* = 0.10, *P* = 0.55) and HbA_1c_ (*r* = 0.24, *P* = 0.13). In a multivariate regression model, between cIMT (dependent) and group (explanatory), only adjustment for S_i_ affected the significance (ß = 0.08, *P* = 0.11) *vs.* (ß = 0.07, *P* < 0.01) for the whole model. No interaction between cIMT, groups and S_i_ was observed.

**Conclusions:**

cIMT is increased and associated with insulin resistance in adolescent, non-obese type 1 diabetic subjects. Although, no conclusions toward a causal relationship can be drawn from current findings, insulin resistance emerges as an important factor reflecting early signs of atherosclerosis in this small cohort.

## Background

Recent advances in diabetes treatment have been successful in postponing the development of microvascular complications through better glycemic control following intensive insulin treatment [1]. In contrast, macrovascular complications have been less affected by this intervention and seem to appear even before onset of hyperglycemia, particularly in patients with type 2 diabetes. In fact, the relative risk for cardiovascular disease (CVD) is even higher in type 1 diabetes, in which juvenile-onset diabetes and its renal complications are modifiers of the natural history of atherosclerosis [2]. Atherosclerosis usually starts with fatty streaks, and can already be seen in childhood [3,4]. Also, early signs of atherosclerosis can be detected by using noninvasive high resolution ultrasonography, measuring the intima-media thickness of the common carotid artery (cIMT), which in turn positively correlates to and predicts CVD [5].

Increased risk for diabetes-related vascular complications is associated with components of the metabolic syndrome, in which insulin resistance might be an important factor [6,7]. However, in the Diabetes Control and Complications Trial type 1 diabetes patients with the highest insulin resistance assessed by estimated glucose disposal rate at baseline had the highest risk of developing both micro-, and macrovascular complications regardless of which treatment group they were randomized to [8]. Moreover, gender differences might occur early in the diabetic state, whereas girls being more insulin resistant, reflected by a higher HbA_1c_ and cholesterol levels, despite increased insulin dose after one year. This gender difference seems to prevail and even increase at time of puberty [9]. The impact of insulin resistance *per se* on CVD is not easy to determine since it is clustering with several other traditional risk factors, *i.e.* hypertension, obesity, elevated triglycerides and low levels of HDL-cholesterol [10]. Nevertheless, it has been evaluated in a mathematical model that preventing insulin resistance would yield as much as 40% of myocardial infarction prevention in young adults, regardless of the other risk factors involved in the metabolic syndrome [10].

To this end, increased cIMT has been demonstrated in adolescents with type 1 diabetes, *i.e.* in association with a blunted endothelial regenerative capacity and low adiponectin levels [11]. A number of studies show increased cIMT in children with the metabolic syndrome and insulin resistance [5,12-16], as well as in adolescent type 2 diabetes [17]. Notwithstanding this, studies on the relationship between insulin resistance and cIMT in young subjects with type 1 diabetes are scarce. Therefore, we sought to investigate early signs of atherosclerosis, measured as cIMT and its relation to insulin sensitivity (S_i_) using hyperinsulinemic euglycemic clamp technique, in a group of adolescent and young adult with type 1 diabetes compared with non-diabetic individuals, all without the metabolic syndrome.

## Methods

### Subjects

Twenty adolescent and young adult type 1 diabetes individuals from the diabetes outpatient clinic at Sachs’ Children’s Hospital, Stockholm, Sweden, were invited to participate in the study. Twenty healthy individuals constituted a control group. The latter were invited from schools from the same area as the diabetic children. Inclusion criteria were: known type 1 diabetes (diabetes group), diabetes duration > 1 year (diabetes group), age 14 – 20 years, willingness to participate in the study, and written informed consent. Exclusion criteria were: any use of oral anti-diabetic agents, lipid lowering medication, ACE/A-II inhibitor treatment and known metabolic syndrome, according to National Cholesterol Education Program Adult Treatment Panel III [18], when three or more criteria were present: 1) Waist circumference >102 cm in men or >88 cm in women; 2) Triglycerides ≥1.7 mmol/l; 3) HDL-cholesterol <1.0 mmol/l, in men, or <1.3 mmol/l, in women; 4) systolic blood pressure (sBP) >130 mmHg or diastolic blood pressure (dBP) >85 mmHg or any use of antihypertensive medication; 5) Fasting plasma glucose ≥6.1 mmol/l (control group). As some discrepancies between how to define the metabolic syndrome occur we also used the definition of the metabolic syndrome set up by WHO [19]. For the participating adolescents under the age of 16 years (*n* = 7) we used the International Diabetes Federation (IDF) definition of metabolic syndrome in children and adolescents for the age group 10–16 years; Central obesity measured as waist circumference ≥90^th^ percentile (or adult cutoff if lower) plus any two of the risk factors; triglycerides ≥1.7 mmol/l, HDL-cholesterol <1.03 mmol/l, sBP ≥130 or dBP ≥85 mmHg, fasting plasma glucose ≥5.6 mmol/l [20]. All participants were evaluated for Tanner stage of puberty at the time of the investigation. The evaluation was based on breast development and information on time of menarche for the girls [21] and testis volume and pubic hair development for the boys [22]. Informed consent was obtained from all participants, when applicable also from both parents. Ethical approval was obtained from the Swedish local ethics committee. The study was conducted according to the principles of the Declaration of Helsinki 1975.

### Study design

This was a single centre, cross-sectional study. The primary endpoint was defined as mean group difference in cIMT and its relation to S_i_, measured by glucose infusion rate (GIR), using hyperinsulinemic euglycemic clamp technique. Secondary endpoints were to explore factors in the metabolic syndrome *i.e.* Body mass index [BMI], waist circumference, sBP, triglycerides, HDL-cholesterol and HbA_1c_ and its relation to cIMT, and cross-sectional intima-media area (CIMA). Subjects were admitted to the metabolic research ward after a 12 hour overnight fast. Participants who were treated with continuous subcutaneous insulin infusion (CSII) were instructed to continue their basal infusion until the test began, and those who were treated with multiple daily injections (MDI) were instructed to take their long acting insulin the day before the test but no fast acting insulin in the morning of the test. Fasting blood tests were drawn for biochemical analysis, urine test for microalbuminuria and a euglycemic hyperinsulinemic clamp was performed. Subsequent measurement of the common carotid artery (cIMT and CIMA) was done using high-resolution ultrasonography. Retinopathy data are collected from routine care screening tests with fundus photographs taken every second year after 10 years of age.

### Euglycemic hyperinsulinemic clamp

The hyperinsulinemic clamp was performed as described by De Fronzo *et al.*[23]. In brief, one intravenous needle was placed in the antecubital vein on the left arm and a second one, in a retrograde fashion, on the back of the right hand. The right hand was kept warm with an electric device (Heated air box set at 55°C, University of Nottingham, U.K) for intermittent sampling of arterialized venous blood. In the left arm needle, human Actrapid insulin (40 mU/m^2^; NovoNordisk A/S, Copenhagen, Denmark) was infused along with 20% dextrose (Fresenius Kabi, Stockholm, Sweden). Based on arterialized blood glucose samples taken from the right hand vein catheter every 5 minutes, the rate of dextrose infusion was adjusted to achieve a blood glucose level of 5.0 mmol/l, during the clamp. Whole-body S_i_ was measured as GIR and calculated from the amount of dextrose infused during the last 30 min of the clamp divided by body weight (kg) and period (min) and expressed as mg/kg/min. The glucose clamp-derived index of S_i_ [S_i_ index; in (10^-4^ dl/kg/ min)/(μU/ml)] was calculated from GIR, corrected for body weight, during the final 30 min as follows: S_i_ index = GIR_ss_/G_ss_ x ΔI_ss_, where GIR_ss_ is the steady state GIR (mg/min), G_ss_ is the steady state blood glucose concentration (mg/dl), and I_ss_ is the difference between basal and steady state plasma insulin concentrations (μU/ml). This calculation is assumed to correct for differences in prevailing glucose and insulin concentrations.

### Blood chemistry

Fasting blood samples were drawn for analyses of HbA_1c_, insulin, C-peptide, total cholesterol, LDL-cholesterol, HDL-cholesterol, triglycerides and hsCRP, using the local clinical chemistry laboratory (Södersjukhuset, Stockholm). Urine analyses measuring albumin and creatinine secretion were done on one urine portion before the clamp and analyzed at the same laboratory. Blood glucose levels were determined by the glucose oxidase method with a glucose analyzer (2300 STAT PLUS; Yellow Springs Instruments, Yellow Springs, OH, USA). Serum insulin and C-peptide levels were analyzed by an immunometric method with monoclonal antibodies (Modular E 170, Roche Diagnostics Scandinavia, Stockholm, Sweden).

### Carotid intima-media thickness (cIMT)

Left and right carotids were examined by one operator using a Siemens Acuson Sequoia^TM^ 512 Ultrasound System (Mountain View, CA, USA) with an 8 MHz linear array transducer. The subject′s head was tilted in order to get the common carotid artery (CCA) just proximal to the bulb placed horizontally across the screen. Magnified pictures were frozen incidentally with the R wave on the electrocardiogram. The cIMT was defined as the distance between the leading edge of the lumen-intima echo and the leading edge of the media-adventitia echo in the far wall. Lumen diameter was defined as the distance between the leading edge of the intima-lumen echo of the near wall and the leading edge of the lumen-intima echo of the far wall [24]. The distal part of the CCA, 5–10 mm proximal to the carotid bulb, was used for measurements of cIMT and lumen diameter. All measurements were performed by one operator, blinded to all other data, using an automated computerized analyzing system [25]. The computer system calculated the average intima-media thickness and lumen diameter of the analyzed section. CIMA was calculated using the formula [(lumen diameter + 2 x IMT)/2]^2^ x 3.14 - (lumen diameter/2)^2^ x 3.14 [26]. cIMT and CIMA were calculated from left and right CCA measurements [(sin + dx)/2]. As cIMT of multiple measurements is most widely used [27], it was chosen as our primary end point measurement.

### Statistics

Results are shown as means ± SD. Comparisons between study groups were made by using Student’s *t* test for independency. Test of normality was conducted with Kolmogorov-Smirnov and Shapiro Wilks test. For those parameters not normally distributed, Mann Whitney test was used for comparison between groups. McNemar’s and sign tests were used for dichotomous variables. Spearman test was used for the correlation data. A stepwise multivariate regression analyses were used for further testing associations between cIMT (dependent) and group (explanatory) regarding atherosclerotic risk factors, *i.e.* S_i_, BMI, waist circumference, sBP, triglycerides, HDL-cholesterol and HbA_1c_. All riskfactors were included and successively excluded in order starting with S_i._ P *<* 0.05 was considered statistically significant. All statistical analyses were performed using PASW/SPPSS Statistics 18 software package.

## Results

### Group characteristics and metabolic parameters

All clinical and biochemical of the study groups are shown in Table 1. All 40 participants were of Caucasian ethnicity. The type 1 diabetic group had mean diabetes duration of 7.3 years. Mean HbA_1c_ in the diabetes group was 74 mmol/mol (DCCT standard = 8.9%). Seven of the type 1 individuals were treated with CSII and 13 with MDI. Those treated with CSII had a mean insulin dose of 0.84 U/kg/day and the ones on MDI 0.94 U/kg/day. There was no difference in HbA_1c_ between CSII and MDI treated diabetics (68 *vs.* 77 mmol/mol, *P* = 0.34). As expected, glucose homeostasis differed between groups; however there were no significant differences in BMI, waist, blood pressure or in lipid profile between type 1 diabetic and healthy groups (Table 1). Mild retinopathy was found in seven diabetics and moderate in one. Statistical analysis showed significant correlation between diabetes duration and retinopathy (β = 0.65, *P* < 0.001). 

**Table 1:** Clinical, biochemical, metabolic and vascular characteristics of the study groups

**Variables**	**Type 1 diabetes**	**Controls**	***P***
Age (yrs)	18 ± 1.5	18 ± 1.7	0.75
Sex (female/male)	8/12	13/7	0.11
Pubertal stage nr (%)			0.28
2	1	0	
4	2	2	
5	17	18	
Smoking (n)	1	4	0.38
Body weight (kg)	71.4 ± 15.7	68.1 ± 11.3	0.54
Height (cm)	174 ± 11	173 ± 8	0.84
BMI (kg/m^2^)	23.5 ± 3.8	22.8 ± 4.0	0.44
Waist circumference (cm)	81.7 ± 14	76.2 ± 9	0.17
sBP (mmHg)	116 ± 12	116 ± 13	0.95
dBP (mmHg)	72 ± 9	73 ± 8	0.83
Diabetes duration yrs (range)	7.3 ± 4 (1–14)	-	
Insulin dose U/kg/day	0.9 ± 0.2	-	
Retinopathy			
None	12	-	
Mild	7	-	
Moderate	1	-	
Microalbuminuria (yes/no)	2/18	0/20	
Fasting plasma glucose (mmol/l)	8.6 ± 3.6	4.6 ± 0.3	
HbA_1c_ (mmol/mol)	74 ± 18.6	35 ± 1.9	
Fasting C-peptide (nmol/l)	0.1 ± 0.4	0.5 ± 0.2	
Total cholesterol (mmol/l)	3.9 ± 0.8	3.6 ± 0.8	0.18
Triglycerides (mmol/l)	0.8 ± 0.3	0.8 ± 0.3	0.87
HDL cholesterol (mmol/l)	1.3 ± 0.3	1.4 ± 0.3	0.36
LDL cholesterol (mmol/l)	2.2 ± 0.9	1.9 ± 0.5	0.13
hsCRP (mg/l)	2.1 ± 2.9	1.8 ± 2.8	0.75
GIR (mg/kg/min)	5.0 ± 2.1	7.1 ± 2.2	<0.01
S_i_ index [(10^-4^ dl/kg/min)/(μU/ml)]	10.2 ± 6.7	14.8 ± 5.7	<0.05
cIMT (mm)	0.52 ± 0.1	0.47 ± 0.1	<0.01
CIMA (mm^2^)	9.92 ± 0.8	8.94 ± 1.3	<0.01
Lumen diameter (mm)	5.58 ± 0.3	5.56 ± 0.4	0.85

*cIMT*, intima-media thickness of the common carotid artery; *CIMA*, cross-sectional intima-media area; *GIR*, glucose infusion rate; S_i_ index (see text for definition); *sBP*, systolic blood pressure; *dBP*, diastolic blood pressure; Pubertal stage is defined as Tanner stage B1-5 for girls and G1-5 for boys, as well as gonadal development.

### Clamp data

The type 1 diabetes group was insulin resistant, demonstrating a significantly lower GIR compared to the non-diabetic group (Table 1). The glucose clamp-derived index of insulin sensitivity (S_i_ index; adjusting for insulin concentration during clamp) was correspondingly lower in the diabetes group compared to the non-diabetic group (Table 1).

### Carotid measurements

The type 1 diabetes individuals had a significantly increased cIMT and CIMA (Table 1). There was no difference in carotid lumen diameter between groups (Table 1).

### Correlation data and regression analysis

**Figure 1:**
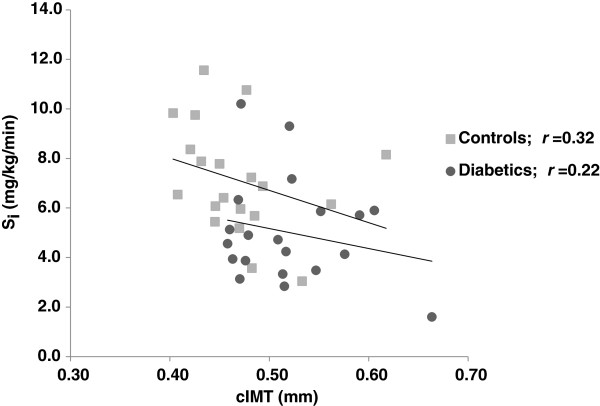
Correlation between cIMT (dependent) and S_**i **_in diabetics and controls, respectively.

S_i_ was negatively associated with cIMT, whereas waist circumference was positively associated with cIMT (Table 2). No such associations were observed for the other atherosclerotic risk factor (Table 2). The correlation, between S_i_ and cIMT, for each group are given in Figure 1. When introducing all the above atherosclerotic risk factors (*i.e.* waist circumference, BMI, sBP, triglycerides, low HDL-cholesterol levels and HbA_1c_), in a multivariate regression model, whereas cIMT and group were the dependent and explanatory factors, respectively, S_i_ abolished the significant association between cIMT and group, with no such effects for the other factors (Table 3). Whenever the S_i_ factor was excluded but including one or more of the other risk factors in multivariate analyses the association between cIMT and group were, again, significant (data not shown). Adjustment for insulin concentration (S_i_ index), in the model, did not change the results (r= 0.021, *P* = 0.07), suggesting that insulin infusion in the clamp not was a confounder. No interaction between groups for the association between S_i_ and cIMT was observed. 

**Table 2:** Correlation between cIMT (dependent) and metabolic factors for atherosclerosis in all subjects (*n* = 40)

**Variables**	***r***	***P***
S_i_	−0.40	<0.01
Waist circumference	0.34	0.11
BMI	0.15	0.03
sBP	0.09	0.58
Triglycerides	0.07	0.66
HDL-cholesterol	0.10	0.55
HbA_1c_	0.24	0.13

*BMI*, Body mass index; *S*_*i*_*,* Insulin sensitivity; *sBP*, systolic blood pressure.

**Table 3:** Stepwise regression analysis of explanatory factors between cIMT (dependent) and group (controls and type 1 diabetes)

**Explanatory**	***β***	***P***
***Included***		
All factors^*^	0.07	<0.01
***Excluded (stepwise in order)***		
S_i_	0.08	0.11
Waist circumference	0.09	0.03
BMI	0.10	<0.01
sBP	0.09	0.01
Triglycerides	0.08	<0.01
HDL-cholesterol	0.08	<0.01
HbA_1c_	0.05	<0.01

^*^S_i_, BMI, Waist circumference, sBP, Triglycerides, HDL-cholesterol and HbA_1c_.

## Discussion

We show that non-obese young adults with type 1 diabetes have early signs of atherosclerosis, as reflected by a significantly increased cIMT concomitant with lower insulin sensitivity, compared with matched non-diabetic individuals. In addition, insulin sensitivity correlated inversely with cIMT, regardless of glycemic control, blood pressure, lipids and BMI.

Young adults with an increased burden of components of the metabolic syndrome have increased cIMT, in whom hypertension and low HDL-cholesterol levels seem to be powerful predictors [16]. Importantly the young type 1 diabetes individuals in the present study were insulin resistant despite any clinical characteristics of the metabolic syndrome and, therefore, enabled us to investigate the correlation between insulin sensitivity *per se* and cIMT. Recently, in attempt to examine the influence of insulin sensitivity on cIMT, young adults were investigated with the clamp technique but no relationship was observed between these factors [13]. This is in contrast to other studies [28-32], as well as the present study. We have no good explanation for this observation, although differences in population, variations in cardiometabolic risk profile and extent of insulin resistance make these studies not easily comparable [33]. At the same time development of factors in the metabolic syndrome seems to be fairly similar in different populations of children and adolescents [34]. Notwithstanding this, our finding is consistent with some previous studies suggesting that insulin resistance is a major component of the metabolic syndrome, beyond glycemic control and clinical characteristics for the metabolic syndrome, in young type 1 diabetes [35-37].

Despite no differences in blood pressure, waist circumference, BMI, plasma lipid levels or smoking habits, cIMT was significantly increased in type 1 diabetic subjects compared to healthy individuals. Clustering of metabolic factors, such as hyperglycemia and insulin resistance, are risk factors suggested to be involved in the progression of cIMT [16,38]. Interestingly, after adjustment of the components in the metabolic syndrome *i.e.* blood pressure, BMI, waist circumference, triglycerides, HDL cholesterol and HbA_1c_, only S_i_, affected group differences. This finding suggests that insulin resistance is the most powerful factor associated to cIMT in the current study. The correlation coefficient, and in particular the *r*^2^ value, suggests that 16% of changes in cIMT can be accounted for by changes in S_i_. In contrast, this correlation was smaller for each group tested suggested being due to the small sample size. There was also no interaction between groups, cIMT and S_i_, supporting the idea that insulin resistance is a single important factor for CVD in a general population [10,39,40].

HbA_1c_ did not correlate with cIMT in the present study. Previous studies suggest that increased cIMT in type 1 diabetes patients is due to diabetes itself, concomitant with an increase in LDL cholesterol [41]. In contrast, long-term follow up studies clearly demonstrate that the progression of cIMT is largely explained by differences in HbA_1c_[42], and that diabetes duration, sBP and BMI could influence the increment in cIMT in childhood and adolescent diabetes [43]. However, neither intensive diabetes treatment, nor levels of HbA_1c_, had any influences on early cIMT changes [44]. This might be explained by the involvement of formation of long-lived advanced glycation end products, highly reactive to the vessel wall, which usually takes several years to develop. Nevertheless, early and intensive insulin treatment in individuals with type 1 diabetes seems to be important to slow cIMT progression independent of other traditional CVD risk factors [42]. This is in contrast with a recent study demonstrating that a high cumulative dose of insulin associates with an increase in cIMT in type 1 diabetes. In an attempt to minimize the effect of insulin resistance as a potential confounder, these researchers investigated type 1 diabetes individuals [45]. We now clearly show that in our currently studied adolescent and young adult type 1 diabetes individuals, who, despite being devoid of any characteristics of the metabolic syndrome, are insulin resistant. The dysregulation of carbohydrate and lipid metabolism ensuing insulin resistance may serve to exacerbate the atherosclerotic progression. In the insulin resistant state, the normal suppression by insulin of free fatty acid release from adipose tissue is impaired so that the characteristic diabetic dyslipidemia occurs, *i.e.* hypertriglyceridemia, low HDL-cholesterol concentrations and elevation of free fatty acids. Defective insulin mediated fatty acid suppression also is suggested to induce insulin resistance in type 1 diabetes individuals [36]. Although our participants had no features of the metabolic syndrome, we cannot entirely exclude that high levels of free fatty acids might explain the association between insulin resistance and cIMT observed. Pro-coagulability and low grade inflammation, often seen in chronic hyperglycemia, can induce insulin resistance [46]. However, as there was no sign of low-grade inflammation in the present study, it is unlikely that heightened inflammatory activity was involved in the cIMT thickening in the diabetic subjects.

### Limitations

The special strengths of the present study are worth emphasizing: only individuals without any clinical features of the metabolic syndrome took part in the study, which therefore allows us to investigate the role of insulin sensitivity *per se* on cIMT. However, certain weaknesses have to be mentioned as well: since this was a cross-sectional study, we cannot draw any conclusions as to causal relationships between insulin resistance and cIMT. We also cannot exclude parameters other than the classical metabolic factors causing linkage between our findings such as non-esterified fatty acids.

## Conclusions

We show that thickening of carotid intima-media, an established surrogate marker of early atherosclerosis, is inversely associated with insulin sensitivity in non-obese adolescent type 1 diabetes. Insulin resistance thus emerges as an important factor reflecting early signs of atherosclerosis in this small cohort. Further studies are warranted to mechanistically explore this finding in detail.

## Abbreviations

sBP: Systolic blood pressure; dBP: Diastolic blood pressure; BMI: Body mass index; CCA: Common carotid artery; cIMT: Intima-media thickness of the common carotid artery; CIMA: Cross-sectional intima-media area; CVD: Cardiovascular disease; GIR: Glucose infusion rate; Si: Insulin sensitivity; CSII: Continuous subcutaneous insulin infusion; MDI: Multiple daily injections.

## Competing interests

The authors declare that they have no competing interests**.**

## Authors’ contributions

All authors designed the study. BR, SR, and TN conducted the study. BR and TN, analyzed, interpreted the data, and wrote the first draft of the manuscript. All authors took part of the final version of the manuscript.
